# Two-Dimensional Perovskite Single-Nanowire Photodetectors

**DOI:** 10.1021/acsphotonics.6c00352

**Published:** 2026-04-20

**Authors:** Avija Ajayakumar, Jeong Hui Kim, Nemanja Ninkovic, Prashant Kumar, Chakkooth Vijayakumar, Leonid Rokhinson, Jianguo Mei, Libai Huang, Letian Dou

**Affiliations:** † Davidson School of Chemical Engineering, 311308 Purdue University, West Lafayette, Indiana 47907, United States; ‡ Chemical Sciences and Technology Division, CSIR-National Institute for Interdisciplinary Science and Technology (NIIST), Thiruvananthapuram 695 019, India; § Academy of Scientific and Innovative Research (AcSIR), Ghaziabad 201 002, India; ∥ Department of Physics and Astronomy, Purdue University, West Lafayette, Indiana 47907, United States; ⊥ Department of Chemistry, Purdue University, West Lafayette, Indiana 47907, United States; # Department of Chemistry, Emory University, 1515 Dickey Drive, Atlanta, Georgia 30322, United States

**Keywords:** perovskite nanowire, microphotodetectors, responsivity, ultralow dark current, thickness dependency

## Abstract

High-performance microphotodetectors require materials that combine strong light–matter interaction, fast charge transport, and ambient stability. Here, we demonstrate single-nanowire devices based on the 2D perovskite (TPA3)_2_PbBr_4_, synthesized via a controlled slow-cooling self-assembly process that yields defect-minimized, anisotropic nanowires with smooth facets. These microphotodetectors exhibit ultralow dark currents (∼10^–15^ A), high responsivity (up to 156 mA W^–1^), and exceptional specific detectivity (∼10^11^ Jones) under near-UV (405 nm) illumination, with rise and fall times in the millisecond regime. The superior detectivity is primarily driven by the suppression of thermal noise through the material’s ultralow dark current, while the millisecond temporal response is governed by high-intensity trap-filling dynamics. The devices maintain stable operation over 4000 s of continuous on/off cycling and show remarkable ambient stability over weeks, attributed to dense crystal packing and robust organic cation layers. Furthermore, the influence of nanowire thickness on the charge collection efficiency is systematically elucidated through optical penetration depth analysis, highlighting design principles for optimizing low-dimensional perovskite photodetectors. This study introduces single 2D perovskite nanowires as a versatile platform for miniaturized, high-performance optoelectronic devices.

## Graphical abstract

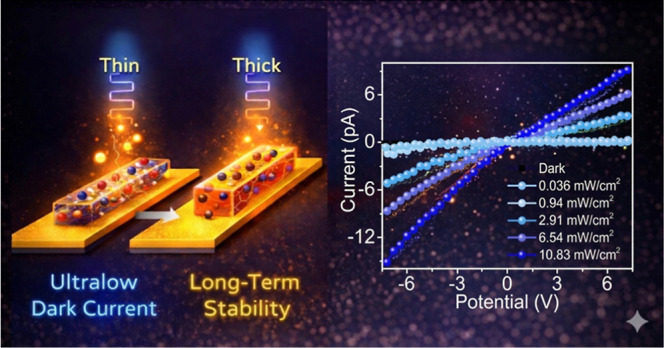

## Introduction

Photodetectors are essential optoelectronic devices that convert incident light into electrical signals, underpinning applications ranging from optical communication and environmental sensing to medical diagnostics and imaging technologies.^1–7^ High-performance photodetectors require a combination of high sensitivity, fast response, low noise, and robust stability.^8–10^ Microphotodetectors, with their compact active areas and shortened carrier transit lengths, provide distinct advantages over larger-area devices, including lower power consumption, faster temporal response, and improved signal-to-noise ratios, making them particularly suitable for miniaturized and integrated optoelectronic systems.^11–14^ In this context, low-dimensional materials, especially two-dimensional (2D) nanowires, have garnered significant attention due to their unique structural and electronic properties.^15–22^ Their large surface-to-volume ratios, quantum confinement effects, and anisotropic charge transport pathways enhance light–matter interaction and carrier dynamics, providing a route toward highly efficient, near-UV photodetection.

Metal halide perovskites have emerged as a transformative class of optoelectronic materials, exhibiting high optical absorption coefficients, tunable band gaps, long carrier diffusion lengths, and facile solution processability.^23–28^ These attributes have enabled perovskite photodetectors with responsivities and detectivities that rival or surpass traditional semiconductor devices.^29–31^ While most studies have focused on thin films and bulk single crystals, low-dimensional perovskite nanostructures, particularly 2D nanowires, offer additional benefits, including directional charge transport, reduced trap densities, and enhanced exciton confinement.^32–34^ Several reports have demonstrated promising photodetection using perovskite nanowires; however, achieving uniform, defect-minimized 2D nanowires remains challenging due to complex crystallization dynamics.^35–39^ Furthermore, ambient stability, particularly for lead-halide perovskites, continues to limit practical device applications.^40–42^ Consequently, there is a pressing need to understand growth mechanisms, optimize device architecture, and improve environmental robustness to fully exploit the potential of perovskite 2D nanowire photodetectors, especially for near-UV light.

While our previous work established the fundamental synthesis and structural properties of (TPA3)_2_PbBr_4_,^43^ the specific carrier transport mechanisms and geometric constraints governing microscale device performance remain largely unexplored. Here, we report a detailed study of microphotodetectors based on single 2D nanowires of (TPA3)_2_PbBr_4_. Unlike previous reports focusing primarily on material discovery, this work systematically investigates the device physics of single-nanowire architectures. The nanowires were synthesized via a carefully controlled slow-cooling self-assembly process, yielding anisotropic, defect-minimized structures with smooth facets, guided by directional hydrogen bonding in the organic layers. The resulting microphotodetectors exhibit ultralow dark currents (∼10^–15^ A), high responsivity (*R*) (up to 156 mA W^–1^), excellent specific detectivity (D*) (∼10^11^ Jones), and fast rise and fall times in the millisecond regime under 405 nm near-UV illumination. We further elucidate the transition from trap-limited to trap-free transport as a function of incident photon flux, explaining the dramatic enhancement in response speed at higher intensities. Devices maintain stable photoresponse over 4000 s and demonstrate robust ambient stability over several weeks, attributed to the dense 2D crystal packing and structural order imparted by bulky organic cations. Systematic studies reveal that thinner nanowires improve charge collection efficiency by enhancing light penetration and minimizing carrier recombination losses. By correlating optical penetration depth with nanowire thickness, we establish critical design principles for optimizing low-dimensional perovskite photodetectors. Our work thus establishes a new platform for high-performance 2D perovskite nanowire microphotodetectors, addressing key challenges in crystal growth, device optimization, and environmental stability, while opening avenues for next-generation near-UV perovskite optoelectronics.

## Results and Discussion

The two-dimensional perovskite (TPA3)_2_PbBr_4_ nanowires were obtained by solution-based crystallization using both floating growth and slow cooling methods. In the floating growth method, a clear, refluxing precursor solution was cooled to ∼70 °C and left undisturbed until crystallization began. In the slow cooling method, the solution temperature was carefully lowered over 72–96 h. Building on our previous synthesis of (TPA3)_2_PbBr_4_, we confirmed that very slow cooling remains essential for obtaining high-quality crystals. Rapid crystallization results in micro- and nanocrystals with poorly defined, pointy end facets caused by uncontrolled one-dimensional growth, whereas the slow self-assembly process consistently yields well-formed, uniform needle-like crystals with smooth facets. This behavior aligns with recent reports demonstrating that directional hydrogen bonding between organic cations serves as a structural template, promoting anisotropic one-dimensional growth in layered perovskites. Specifically, the TPA3^+^ cations in (TPA3)_2_PbBr_4_ form parallel one-dimensional chains within the organic layers, connected by periodic COOH–COOH dimers with hydrogen-bond distances of approximately 1.78 Å along the (010) crystallographic direction. These strong, directional noncovalent interactions drive the anisotropic, needle-like self-assembly of the two-dimensional perovskite into long nanowires with an average aspect ratio of ∼28.9. As illustrated in Figure 1a,b, only the crystals grown via slow cooling exhibit clean, flat end facets, confirming the necessity of slow crystallization to achieve uniform one-dimensional morphology.

**Figure 1:**
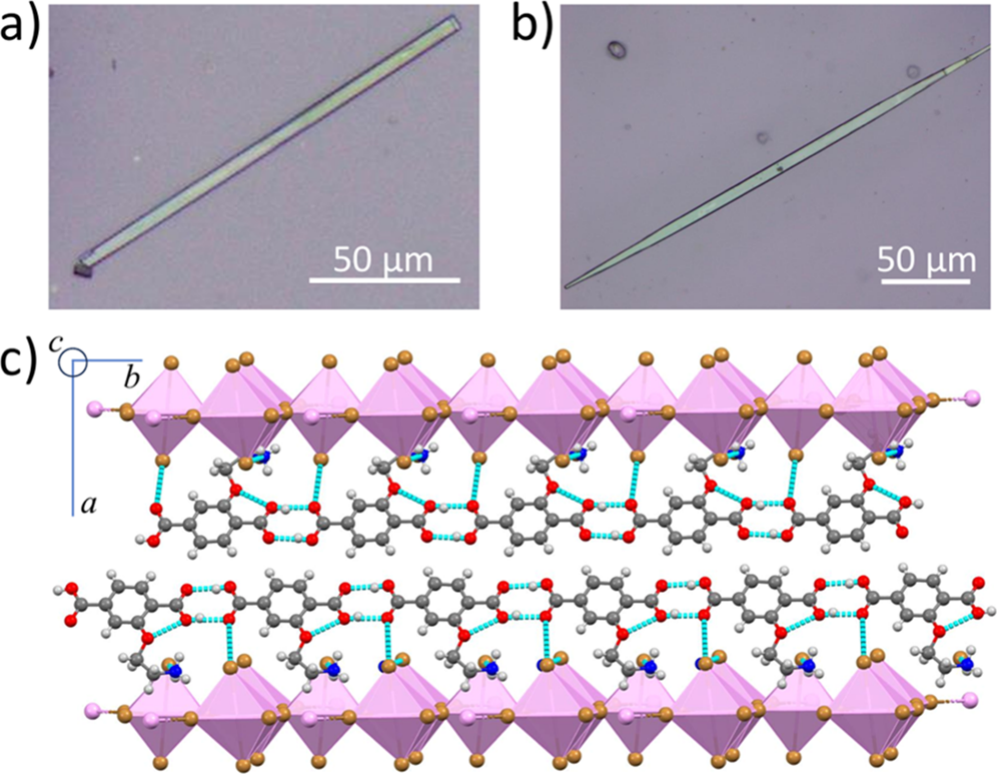
(TPA3)_2_PbBr_4_: (a) slow assembled crystal; (b) fast-assembled crystal. (c) Single-crystal image of (TPA3)_2_PbBr_4_ showing the 1D hydrogen-bonding chains in the crystal lattice (Pb: pink, Br: brown, C: dark gray, H: light gray, O: red, N: blue, and blue dashed line indicates hydrogen bond).

Single-crystal X-ray diffraction analysis (Figure 1c) confirms that (TPA3)_2_PbBr_4_ forms a layered structure of corner-sharing PbBr_6_ octahedra separated by organic TPA3 cations, consistent with our previous report. Optical spectroscopy of the large single crystals (Figure S1, Supporting Information) reveals negligible narrow excitonic features; instead, the emission is dominated by a broad, Stokes-shifted photoluminescence band extending over several 100 meV. Such emission is characteristic of low-dimensional halide perovskites and is commonly attributed to self-trapped excitons or defect-related states, facilitated by strong electron–phonon coupling within the layered Pb–Br lattice. These observations indicate that (TPA3)_2_PbBr_4_ nanowires retain the structural and optical features of 2D perovskites, effectively behaving as low-dimensional quantum wells with pronounced STE-related luminescence.

To measure charge transport, we fabricated photodetector devices by using individual nanowires. Gold (Au) electrodes (45 nm thick) were patterned on Si/SiO_2_ substrates by electron beam lithography, photolithography, and metal evaporation. Subsequently, synthesized nanowires were transferred onto these electrodes through a deterministic dry transfer method employing a polydimethylsiloxane (PDMS) stamp. The nanowire transfer process is detailed in the Supporting Information. Figure 2a shows an optical micrograph of a typical device, consisting of a single (TPA3)_2_PbBr_4_ nanowire bridging two Au pads (SEM images of devices with nanowires of different thicknesses are provided in Figure S2, Supporting Information). Electrical measurements were performed by contacting the Au electrodes; the linear and symmetric current–voltage (*I*–*V*) behavior (Figure 2b) confirms that the contacts are essentially Ohmic (a linear *I*–*V* is expected when there is no significant Schottky barrier at the metal–semiconductor interface).

**Figure 2:**
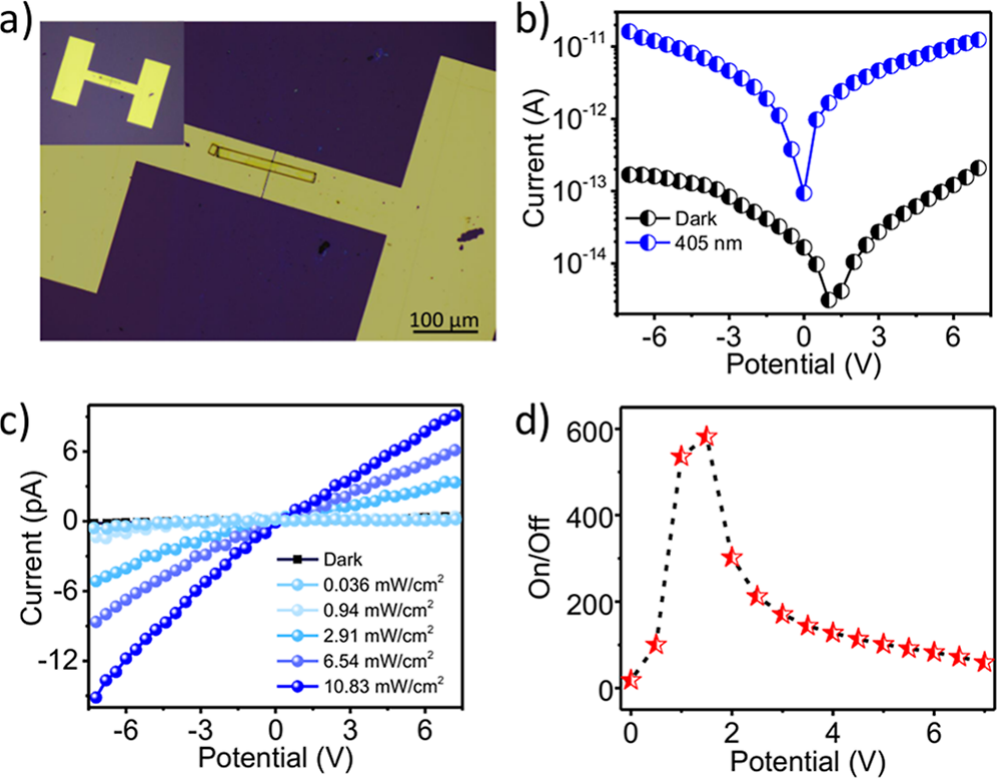
(a) Microscopic image showing a single nanowire bridging the Au electrodes in the microphotodetector device (inset: full device image). (b) Current–voltage (*I*–*V*) characteristics of a (TPA3)_2_PbBr_4_ nanowire measured under dark conditions and under 405 nm illumination (*P*_in_ = 6.54 mW/cm^2^). (c) *P*_in_-dependent *I*–*V* characteristics. (d) Variation of on/off ratio with respect to bias voltage.

Under dark conditions (no illumination) the device shows an ultralow dark current, on the order of 10^–15^ A at 1.5 V bias. Such low leakage reduces noise and enhances sensitivity. When illuminated with 405 nm near-UV laser (photon energy above the ∼3 eV band gap), the current rises sharply. The photodetector responds strongly across the 400–420 nm range but is almost insensitive to wavelengths above 420 nm, demonstrating selective near-UV detection. As shown in Figure 2b, at 1.5 V bias the dark current (∼9.5 × 10^–15^ A) increases to ∼9.98 × 10^–13^ A under 405 nm illumination (6.54 mW/cm^2^), while at 7 V bias, the photocurrent reaches ∼6.2 × 10^–12^ A.

To gain further insight into device performance, the photoresponse was measured across a range of illumination intensities (*P*_in_ = 0.036–10.80 mW/cm^2^), as shown in Figure 2c. The photocurrent increases monotonically with light intensity as higher photon flux generates more charge carriers, including contributions from deep trap states, thereby enhancing the overall photocurrent. The bias-dependent on/off ratio, a key figure of merit for photodetectors, was also evaluated (Figure 2d). The device achieves a maximum on/off ratio of 6 × 10^2^ at 2 V bias, calculated from the *I*–*V* characteristics, exceeding values reported in comparable lead-halide perovskite nanowire devices. Such high on/off contrast reflects excellent signal-to-noise performance under illumination and underscores the superior photodetection capability of the single-nanowire architecture. Overall, the (TPA3)_2_PbBr_4_ nanowire exhibits a pronounced photoresponse with ultralow dark current and strong photocurrent amplification under 405 nm near-UV excitation, highlighting its potential for high-performance, miniaturized photodetector applications.

Time-resolved photoresponse measurements were performed under pulsed 405 nm near-UV illumination to assess the dynamic performance of the device at different bias voltages (Figure 3a,b). At low bias voltages (<5 V), the photocurrent is present but exhibits minor fluctuations (Figure S3), reflecting incomplete charge collection. When the bias is increased to ≥5 V, the response becomes stable and highly reproducible, indicating efficient carrier extraction. Interestingly, further increasing the bias to 6–7 V does not significantly enhance the steady-state photocurrent (Figure 3a,b), suggesting that charge collection efficiency is already maximized at 5 V, and additional electric field primarily increases the dark current. Under repeated on/off illumination cycles at 5 V bias, the device demonstrates robust operational stability over 4000 s (several thousand cycles), as shown in Figure 3c. While minor fluctuations are observed in the peak photocurrent during extended cycling, these are attributed to transient thermal effects or local pyroelectric responses under continuous high-intensity near-UV excitation rather than chemical or structural degradation of the perovskite lattice. The preservation of the baseline dark current and the rapid restoration of peak photocurrent across thousands of cycles underscore the long-term reliability of single (TPA3)_2_PbBr_4_ nanowire-based microphotodetectors.

**Figure 3:**
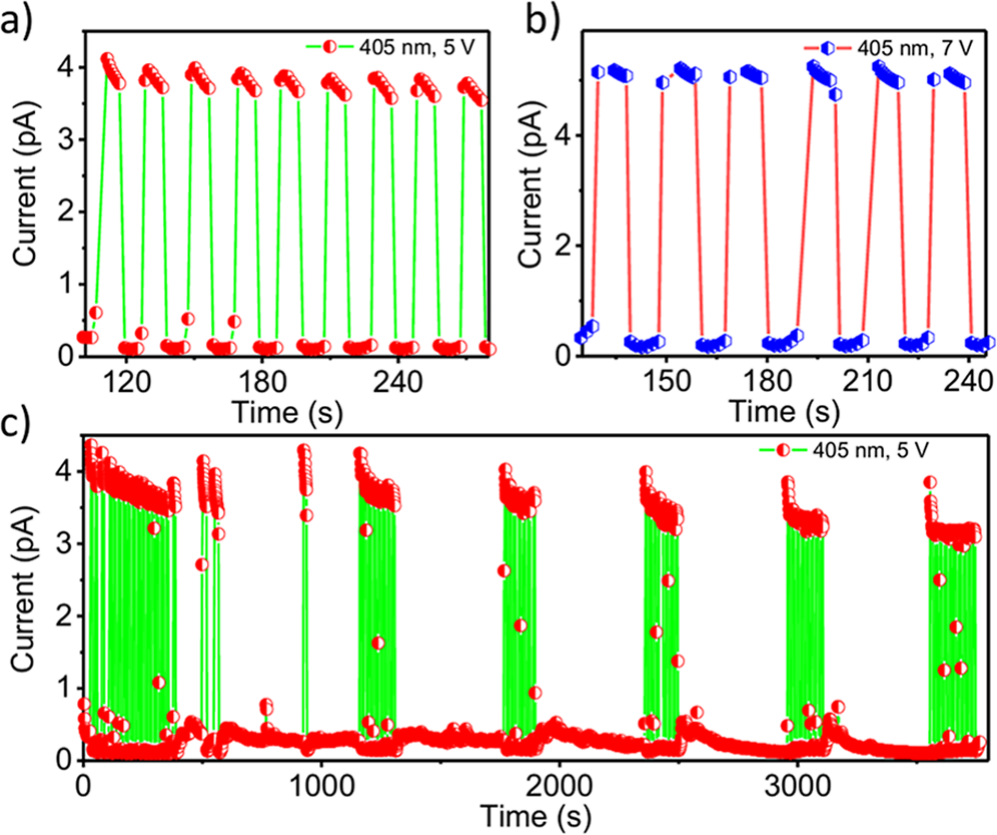
(a) Temporal photoresponse under 405 nm illumination at a bias voltage of (a) 5 V and (b) 7 V. (c) Cyclic stability study of the device over 4000 s.

The temporal response of the photodetector was evaluated through its rise and fall times (*t*_r_ and *t*_f_), defined as the intervals for photocurrent to change between 10% and 90% of its maximum value. Notably, the device exhibits a strong dependence on illumination intensity. At 0.94 mW cm^–2^, the rise time was 0.14 s and the fall time was 0.17 s (Figure 4a). Increasing the intensity to 10.83 mW cm^–2^ accelerated the response dramatically, reducing rise and fall times to 16 and 22 ms, respectively. This pronounced enhancement is attributed to trap-filling dynamics within the 2D perovskite nanowires.^44,45^ At lower intensities, photogenerated carriers are frequently captured by shallow trap states, which slows the temporal kinetics of carrier transport. As the incident photon flux increases, these trap states become saturated (filled), allowing subsequent carriers to move more freely through the lattice. This transition from trap-limited to trap-free transport facilitates faster carrier generation and extraction, resulting in the observed millisecond-scale response times.

**Figure 4:**
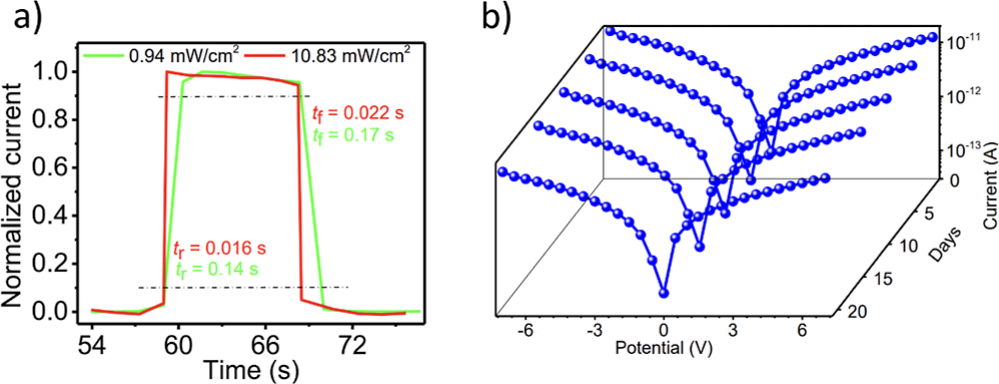
(a) Response speed (rise and fall time) of the device at two different illumination intensities of 0.94 mW/cm^2^ and 10.83 mW/cm^2^. (b) *I*–*V* characteristics measured at 5 day intervals (under illumination).

It is important to note that no plateau in the response time is observed within the illumination range accessible in our measurements. This is because the available photon flux remains below the regime required for complete saturation of both shallow and deep trap states. Similar 2D perovskite systems reported in the literature show that response-time saturation typically occurs only at substantially higher excitation intensities,^46–48^ well beyond the safe operating window of our nanowire devices. Consequently, the rise and fall times continue to decrease with increasing illumination in our experiments, consistent with trap-filling-dominated dynamics rather than full trap saturation.

The device also demonstrates exceptional ambient stability, a critical requirement for lead-halide perovskites. Stored under standard laboratory conditions, the photocurrent showed only minor reduction after 20 days, and the *I*–*V* characteristics and on/off ratio remained essentially unchanged even after 40 days (Figures 4b and S4). This robust stability arises from the combined effect of the bulky TPA3 cations and dense 2D crystal packing, which protect the inorganic lattice from environmental degradation while preserving an efficient charge transport.

The performance of the photodetector was systematically evaluated by extracting key figures of merit, namely responsivity (*R*), specific detectivity (D*), and external quantum efficiency (EQE), as summarized in Figure 5. These parameters are critical for assessing the device’s efficiency and sensitivity. Responsivity quantifies the effectiveness of converting incident optical power into an electrical signal, while detectivity reflects the ability to discern weak optical inputs by incorporating both responsivity and noise considerations. EQE represents the fraction of incident photons successfully converted into charge carriers contributing to the photocurrent.

**Figure 5:**
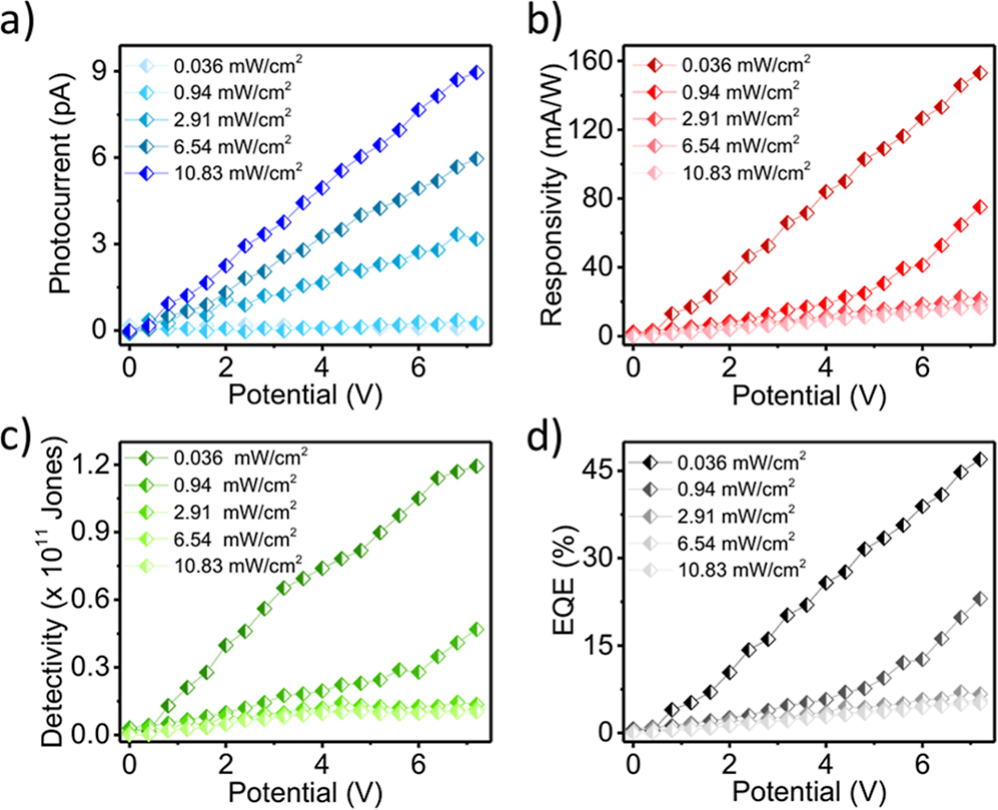
Bias voltage and illumination intensity dependent (a) photocurrent; (b) responsivity; (c) detectivity; and (d) EQE.

Mathematically, the responsivity is given by

$$R=I_{ph}/P_{in}A$$

where *I*_ph_ is the photocurrent, *P*_in_ is the incident optical power, and *A* is the effective illuminated area. Since *R* is directly proportional to the photocurrent and inversely proportional to the incident light intensity, its variation with applied bias provides valuable insights into charge carrier generation, transport, and recombination dynamics.

The specific detectivity, which accounts for sensitivity while considering noise sources, can be expressed as

$$D^{*}=RA^{1/2}/{\left(2eI_{dark}\right)}^{1/2}$$

where *I*_dark_ is the dark current and *e* is the elementary charge. This simplified approach assumes that shot noise from dark current is the dominant noise source. The EQE is directly related to *R* through the incident photon flux and follows the same general bias and intensity dependence.

Figure 5 illustrates the bias-dependent photocurrent, *R*, D*, and EQE values under varying illumination intensities. The photocurrent increases with both bias voltage and light intensity as higher photon flux generates more photocarriers. However, *R* and D* increase with the bias voltage but decrease with the illumination intensity. This inverse trend with light intensity arises because although photocurrent increases, it does not scale proportionally to the input optical power, likely due to carrier recombination or trap state saturation, resulting in a reduced responsivity. As D* and EQE are directly related to *R*, they also exhibit a similar decrease with higher light intensity but an increase with applied bias.

From Figure 5a, the maximum photocurrent of 9.2 pA is obtained at a 7 V bias under 10.83 mW cm^–2^ illumination. The peak responsivity and detectivity values, obtained from Figure 5b,c, are 156 mA W^–1^ and 1.26 × 10^11^ Jones, respectively, while the highest EQE recorded is 46% (Figure 5d).

To further contextualize the performance of the (TPA3)_2_PbBr_4_ single-nanowire photodetector, we compared our results with several recently reported perovskite-based nanowire and single-crystal devices (Table 1).^46–54^ While some 3D perovskites, such as MAPbI_3_ and CsPbBr_3_, exhibit higher absolute responsivities due to internal gain mechanisms, they often suffer from significant dark currents (ranging from 10^–9^ to 10^–12^ A) and slower response times. In contrast, our (TPA3)_2_PbBr_4_ device stands out by delivering an ultralow dark current of ∼10^–15^ A, which is several orders of magnitude lower than most reported values. This suppression of the noise floor allows for high specific detectivity (∼10^11^ Jones) despite the moderate responsivity. Furthermore, our device maintains a competitive millisecond-scale response speed (16/22 ms), whereas many high-gain devices operate in the regime of hundreds of milliseconds. This balance of ultralow noise, rapid kinetics, and high ambient stability demonstrates that the (TPA3)_2_PbBr_4_ single-nanowire architecture is a superior candidate for high-contrast near-UV photodetection in miniaturized systems.

**Table 1:** Comparison of Performance Metrics for Various Perovskite-Based Photodetectors

material	architecture	dark current (A)	responsivity (mA/W)	detectivity (Jones)	response time (ms)	refs
CsSnI_3_	NWs	dark current	9.9	7.2 × 10^8^	446/534	^49^
CsPbI_3_	NWs	-	6.7	1.57 × 10^8^	292/234	^50^
MAPbI_3_	NWs	-	30	10^10^	20.47/13.81	^51^
CsPbBr_3_	single MW	10^–12^	118 × 10^3^	10^12^	40	^52^
CsPbBr_3_	NWs	10^–10^	7.66 × 10^3^	10^12^	275/550	^53^
(PEA)_2_PbBr_4_	single crystal	10^–13^	31.48	10^13^	0.41/0.37	^54^
(PEA)_2_PbI_4_	single NW	10^–11^	1.9 × 10^3^	10^12^	69.6/69.5	^55^
CsPbI_3_	NWs	10^–9^	350	10^10^	-	^56^
CsPb_*x*_Sn_1–*x*_(Br_*y*_I_1–*y*_)_3_	NWs	10^–10^	-	10^10^	4.25/4.82	^57^
(BA)_2_(MA)Pb_2_I_7_	single crystal	10^–12^	-	10^11^	125/74	^58^
(BA)_2_PbI_4_/(BA)_2_(MA)Pb_2_I_7_	heterostructure	10^–12^	11.5 × 10^3^	10^11^	150/170	^59^
**(TPA3)**_**2**_ **PbBr**_**4**_	**single NW**	**10** ^**–15**^	**156**	**10** ^**11**^	**16/22**	**this work**

We fabricated and tested photodetectors using nanowires of different thicknesses (4, 6, and 10 μm) to assess how geometry influences performance (Figure 6). All devices showed low dark current, but notably the thicker wires had higher dark current values. This can be understood as arising from a greater number of intrinsic carriers within the larger crystal volume. Conversely, the thinner wires produced higher photocurrents and thus higher responsivities (Figure 6a,b). In fact, both *R* and D* decreased with increasing thickness. We attribute this trend to the relationship between optical penetration depth (1/α) and carrier collection efficiency. In our dry-transfer device geometry, the nanowire sits atop the electrode. For a thick wire, near-UV light is strongly absorbed near the top surface, but the photons do not penetrate effectively to the bottom layer in direct contact with the electrode. Consequently, photocarriers generated deep within the wire must traverse a longer distance—and encounter more recombination centers or traps—before reaching the contact. In a thinner wire (e.g., 4 μm), the entire cross-section is illuminated, and photocarriers are generated in close proximity to the Au electrodes, enabling more efficient collection and reduced recombination losses. A schematic (Figure 6c) illustrates that when the base of the nanowire receives light, charge migration to the contact is optimized. Therefore, thinner (TPA3)_2_PbBr_4_ nanowires serve as a critical design parameter for maximizing sensitivity in this lateral architecture.

**Figure 6:**
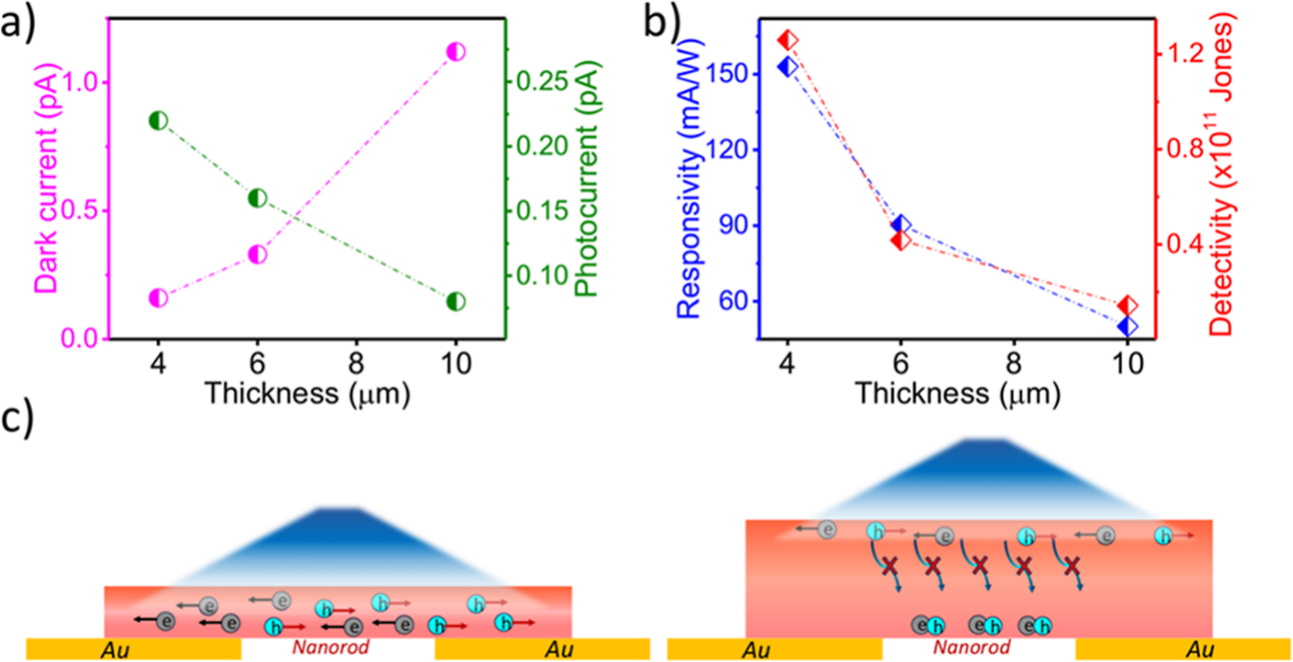
Nanowire thickness dependence of (a) dark current and photocurrent; (b) responsivity and detectivity; (c) schematic illustrating photocurrent generation and transport in thinner versus thicker nanowires.

It is worth emphasizing that microphotodetectors based on single 2D perovskite nanowires remain scarcely reported; here, we provide a comprehensive evaluation that bridges the gap between material properties and device physics. The (TPA3)_2_PbBr_4_ single-nanowire architecture demonstrates a stable and dynamic photoresponse, characterized by an exceptionally low dark current (∼10^–15^ A), high responsivity (*R*), and superior specific detectivity (D*). As summarized in Table 1, our device stands out among recently reported perovskite-based photodetectors by offering an ultralow noise floor that is several orders of magnitude lower than most 3D and 2D counterparts. This suppression of dark current allows for high-contrast near-UV sensing even with moderate responsivity.

Furthermore, our investigation reveals that nanowire thickness is a governing design parameter rather than just a morphological trait. By systematically employing thinner wires, we addressed the challenge of light–electrode coupling, ensuring that the optical penetration depth aligns with the charge extraction region for maximized performance. Finally, the robust ambient stability—maintaining functionality for weeks in air—stems from the dense 2D crystal packing and the use of bulky, hydrophobic organic cations that inherently suppress environmental degradation pathways. These findings establish (TPA3)_2_PbBr_4_ nanowires as a viable, high-performance platform for miniaturized, integrated near-UV optoelectronics.

## Conclusion

In summary, we report the first microphotodetector based on single 2D perovskite (TPA3)_2_PbBr_4_ nanowires, delivering a combination of ultralow dark currents (∼10^–15^ A), high responsivity (156 mA W^–1^), and exceptional specific detectivity (∼10^11^ Jones). The devices exhibit selective near-UV photoresponse and stable performance over 4000 s of repeated on/off cycling and several weeks under ambient conditions, demonstrating superior environmental robustness attributed to the dense crystal packing and hydrophobic organic cation layers. Beyond material demonstration, this work provides critical insights into the device physics of 2D perovskite nanowires. We elucidated the intensity-dependent temporal response, where a transition from trap-limited to trap-free transport enables millisecond-scale rise and fall times under high photon flux. Furthermore, systematic studies establish nanowire thickness as a governing factor for charge collection efficiency. By correlating optical penetration depth with carrier transit distance, we demonstrate that thinner nanowires minimize recombination losses and maximize light–electrode coupling. These findings distinguish our work from fundamental crystal synthesis by providing a systematic framework for geometric optimization in microscale optoelectronics. This study establishes single 2D perovskite nanowires as a high-performance, stable platform, paving the way for next-generation, miniaturized near-UV perovskite photodetectors.

## Supplementary Material



## Notes

Microscopy images, PL spectra, SEM images, photoresponse measurements, stability data, and full experimental procedures (PDF)

## References

[1] Li Y., He X., Chen S.-C., Zhao N. (2025). Intelligent Photodetectors: Post
manufacturing Tunability toward Enhanced Performance and Advanced
Functions. Chem. Rev..

[2] Zhou Y., Fei C., Uddin M. A., Zhao L., Ni Z., Huang J. (2023). Self-powered
perovskite photon-counting detectors. Nature.

[3] Chen X., Zhu Y., Xu Y., Rao M., Pang P., Zhang B., Xu C., Ni W., Li G., Wu J., Li M., Chen Y., Geng Y. (2025). Design of
Ultra-Narrow Bandgap Polymer
Acceptors for High-Sensitivity Flexible All-Polymer Short-Wavelength
Infrared Photodetectors. Angew. Chem., Int.
Ed..

[4] Kong W.-Y., Wu G.-A., Wang K.-Y., Zhang T.-F., Zou Y.-F., Wang D.-D., Luo L.-B. (2016). Graphene-β-Ga_2_O_3_ Heterojunction
for Highly Sensitive Deep UV Photodetector
Application. Adv. Mater..

[5] Li Z., Li Z., Zuo C., Fang X. (2022). Application of Nanostructured
TiO_2_ in UV Photodetectors: A Review. Adv.
Mater..

[6] Fan P., Chettiar U. K., Cao L., Afshinmanesh F., Engheta N., Brongersma M. L. (2012). An invisible
metal-semiconductor
photodetector. Nat. Photonics.

[7] Li S., Zhang Y., Yang W., Liu H., Fang X. (2020). 2D Perovskite
Sr_2_Nb_3_O_10_ for High-Performance UV
Photodetectors. Adv. Mater..

[8] Yan F., Wei Z., Wei X., Lv Q., Zhu W., Wang K. (2018). Toward High-Performance
Photodetectors Based on 2D Materials: Strategy on Methods. Small Methods.

[9] Huo N., Konstantatos G. (2018). Recent Progress
and Future Prospects of 2D-Based Photodetectors. Adv. Mater..

[10] Xie C., Yan F. (2017). Flexible Photodetectors
Based on Novel Functional Materials. Small.

[11] Luo Y., Dong Z., Chen Y., Zhang Y., Lu Y., Xia T., Wang L., Li S., Zhang W., Xiang W., Shan C., Guo H. (2019). Self-powered NiO@ZnO-nanowire-heterojunction
ultraviolet micro-photodetectors. Opt. Mater.
Express.

[12] Ma T., Xue N., Muhammad A., Fang G., Yan J., Chen R., Sun J., Sun X. (2024). Recent Progress in Photodetectors: From Materials to
Structures and Applications. Micromachines.

[13] Yu Y., Wang W., Li W., Wang G., Wang Y., Lu Z., Li S., Zhao W., Li Y., Liu T., Yan X. (2022). Photodetectors Based on Micro-nano Structure Material. Front. Chem..

[14] Xu H., Ai J., Deng T., Ruan Y., Sun D., Liao Y., Cui X., Tian P. (2025). Recent Progress in GaN-Based High-Bandwidth Micro-LEDs
and Photodetectors for High-Speed Visible Light Communication. Photonics.

[15] Feng J., Gong C., Gao H., Wen W., Gong Y., Jiang X., Zhang B., Wu Y., Wu Y., Fu H., Jiang L., Zhang X. (2018). Single-crystalline
layered metal-halide
perovskite nanowires for ultrasensitive photodetectors. Nat. Electron..

[16] Zhao Y., Qiu Y., Feng J., Zhao J., Chen G., Gao H., Zhao Y., Jiang L., Wu Y. (2021). Chiral 2D-Perovskite
Nanowires for Stokes Photodetectors. J. Am.
Chem. Soc..

[17] Lee Y. H., Lee W.-J., Lee G. S., Park J. Y., Yuan B., Won Y., Mun J., Yang H., Baek S.-D., Lee H., Oh J. H., Pennycook T. J., Kim G., Mei J., Dou L. (2025). Large-Scale
2D Perovskite Nanocrystals Photodetector Array via Ultrasonic
Spray Synthesis. Adv. Mater..

[18] Yan S.-S., Kong Y.-C., Zhang Z.-H., Wu Z. S., Lian Z. D., Zhao Y. P., Su S. C., Li L., Wang S. P., Ng K. W. (2022). Enhanced Optoelectronic Performance Induced by Ion Migration in Lead-Free
CsCu_2_I_3_ Single-Crystal Microrods. ACS Appl. Mater. Interfaces.

[19] Malik M., Iqbal M. A., Choi J. R., Pham P. V. (2022). 2D Materials
for
Efficient Photodetection: Overview, Mechanisms, Performance and UV-IR
Range Applications. Front. Chem..

[20] Odebowale A. A., Berhe A. M., Somaweera D., Wang H., Lei W., Miroshnichenko A. E., Hattori H. T. (2025). Advances in 2D Photodetectors: Materials,
Mechanisms, and Applications. Micromachines.

[21] Spies M., Monroy E. (2019). Nanowire photodetectors based on wurtzite semiconductor
heterostructures. arXiv.

[22] Zhang W., Xiang Z., Ma T., Bian B., Liu J., Wu Y., Liu Y., Shang J., Li R.-W. (2025). Self-supported β-Ga_2_O_3_ nanowires and for stretchable solar-blind UV
photodetectors. Sci. Rep..

[23] Jena A. K., Kulkarni A., Miyasaka T. (2019). Halide Perovskite Photovoltaics:
Background, Status, and Future Prospects. Chem.
Rev..

[24] Dong H., Ran C., Gao W., Li M., Xia Y., Huang W. (2023). Metal Halide
Perovskite for next-generation optoelectronics: progresses and prospects. eLight.

[25] Ahmadi M., Wu T., Hu B. (2017). A Review on
Organic-Inorganic Halide Perovskite Photodetectors:
Device Engineering and Fundamental Physics. Adv. Mater..

[26] Manser J. S., Christians J. A., Kamat P. V. (2016). Intriguing Optoelectronic Properties
of Metal Halide Perovskites. Chem. Rev..

[27] Cao F., Li L. (2021). Progress of Lead-Free
Halide Perovskites: From Material Synthesis
to Photodetector Application. Adv. Funct. Mater..

[28] Ajayakumar A., Muthu C., Basavarajappa M. G., Dev A. V., Nishikubo R., Chakraborty S., Saeki A., Dou L., Vijayakumar C. (2024). Zero-Dimensional
Tin Halide Perovskite with Long Charge Carrier Lifetime and Anisotropic
Photoconductivity for Selective Deep-UV Photodetection. Adv. Funct. Mater..

[29] Nodari D., Qiao Z., Furlan F., Sandberg O. J., Vandewal K., Gasparini N. (2025). Towards high and reliable specific detectivity in visible
and infrared perovskite and organic photodiodes. Nat. Rev. Mater..

[30] Ke K., Gao Y., Meng J., He Y., Deng B., Huang H., Zhang S.-W., Zhang K., Xu Z., Li H., Yao X., Ye Z., Song L., Shu C., Yang S., Qin N., Fu H. Y., Yip H.-L., Kang F. (2025). High-Performance
Quasi-2D Sn-Pb Perovskite Photodetectors for High-Fidelity Image Sensing
and Optical Communication. Adv. Funct. Mater..

[31] Yadav S. N. S., Hanmandlu C. C., Patel D. K., Singh R. K., Chen C.-Y., Wang Y.-Y., Chu C.-W., Liang C.-T., Lin C.-T., Lu Y.-J., Yen T.-J. (2023). Enhanced Photoresponsivity
of Perovskite
QDs/Graphene Hybrid Gate-Free Photodetector by Morphologically Controlled
Plasmonic Au Nanocrystals. Adv. Opt. Mater..

[32] Zhu H., Fu Y., Meng F., Wu X.-Y., Gong Z., Ding Q., Gustafsson M. V., Trinh M. T., Jin S., Zhu X. Y. (2015). Lead halide
perovskite nanowire lasers with low lasing thresholds and high quality
factors. Nat. Mater..

[33] Zhu T., Gong X. (2021). Low-dimensional perovskite materials and their optoelectronics. InfoMat.

[34] Pan D., Fu Y., Spitha N., Zhao Y., Roy C. R., Morrow D. J., Kohler D. D., Wright J. C., Jin S. (2021). Deterministic fabrication
of arbitrary vertical heterostructures of two-dimensional Ruddlesden-Popper
halide perovskites. Nat. Nanotechnol..

[35] Guan Y., Zhang C., Liu Z., Zhao Y., Ren A., Liang J., Hu F., Zhao Y. (2022). Single-Crystalline
Perovskite p-n Junction Nanowire Arrays for Ultrasensitive Photodetection. Adv. Mater..

[36] Tang Y., Xiao B., Wu D., Zhou H. (2025). Multi-functional PbI_2_ enables self-driven perovskite nanowire
photodetector with
ultra-weak light detection ability. J. Semicond..

[37] Castillo-Seoane J., Contreras-Bernal L., Rojas T. C., Espinós J. P., Castro-Méndez A.-F., Correa-Baena J.-P., Barranco A., Sanchez-Valencia J. R., Borras A. (2024). Highly Stable Photoluminescence
in Vacuum-Processed Halide Perovskite Core-Shell 1D Nanostructures. Adv. Funct. Mater..

[38] Feng M., Byun J., Li Z., Xie Z., Lu W., Wen X., Ding L., Wu T., Jamshaid S., Götz K., Li C., Peng Z., Hu H., Tian J., Elia J., Unruh T., Halik M., Xue D.-J., Osvet A., Brabec C. J. (2025). Overcoming size limits with dynamic templates enabling
large area single crystal nanowire arrays for photodetectors. Nat. Commun..

[39] Kuo M.-Y., Spitha N., Hautzinger M. P., Hsieh P.-L., Li J., Pan D., Zhao Y., Chen L.-J., Huang M. H., Jin S., Hsu Y.-J., Wright J. C. (2021). Distinct Carrier Transport Properties
across Horizontally vs Vertically Oriented Heterostructures of 2D/3D
Perovskites. J. Am. Chem. Soc..

[40] Zhao X., Park N.-G. (2015). Stability Issues
on Perovskite Solar Cells. Photonics.

[41] Li D., Liao P., Shai X., Huang W., Liu S., Li H., Shen Y., Wang M. (2016). Recent progress on stability issues
of organic-inorganic hybrid lead perovskite-based solar cells. RSC Adv..

[42] Chowdhury T. A., Bin Zafar M. A., Sajjad-Ul Islam M., Shahinuzzaman M., Islam M. A., Khandaker M. U. (2023). Stability of perovskite solar cells:
issues and prospects. RSC Adv..

[43] Shao W., Kim J. H., Simon J., Nian Z., Baek S.-D., Lu Y., Fruhling C. B., Yang H., Wang K., Park J. Y., Huang L., Yu Y., Boltasseva A., Savoie B. M., Shalaev V. M., Dou L. (2024). Molecular
Templating
of Layered Halide Perovskite Nanowires. Science.

[44] Rose A. (1955). Space-Charge-Limited
Currents in Solids. Phys. Rev..

[45] Labanti C., Wu J., Shin J., Limbu S., Yun S., Fang F., Park S. Y., Heo C.-J., Lim Y., Choi T., Kim H.-J., Hong H., Choi B., Park K.-B., Durrant J. R., Kim J.-S. (2022). Light-intensity-dependent
photoresponse
time of organic photodetectors and its molecular origin. Nat. Commun..

[46] Fang H.-H., Adjokatse S., Shao S., Even J., Loi M. A. (2018). Long-Lived
Hot-Carrier Light Emission and Large Blue Shift in Formamidinium Tin
Triiodide Perovskites. Nat. Commun..

[47] Deng S., Shi E., Yuan L., Jin L., Dou L., Huang L. (2020). Long-Range
Exciton Transport and Slow Annihilation in Two-Dimensional Hybrid
Perovskites. Nat. Commun..

[48] Yakunin S., Sytnyk M., Kriegner D., Shrestha S., Richter M., Matt G. J., Azimi H., Brabec C. J., Stangl J., Kovalenko M. V., Heiss W. (2015). Detection of X-ray Photons by Solution-Processed
Lead Halide Perovskites. Nat. Photonics.

[49] Zhou H., Tang X., Gao Z. (2022). Lead-less perovskite
alloy nanowire
photodetector with high performance. Colloid
Interface Sci. Commun..

[50] Waleed A., Tavakoli M. M., Gu L., Hussain S., Zhang D., Poddar S., Wang Z., Zhang R., Fan Z. (2017). All Inorganic
Cesium Lead Iodide Perovskite Nanowires with Stabilized Cubic Phase
at Room Temperature and Nanowire Array-Based Photodetectors. Nano Lett..

[51] Gu L., Tavakoli M. M., Zhang D., Zhang Q., Waleed A., Xiao Y., Tsui K.-H., Lin Y., Liao L., Wang J., Fan Z. (2016). 3D Arrays of 1024-Pixel
Image Sensors
based on Lead Halide Perovskite Nanowires. Adv.
Mater..

[52] Gui P., Chen Z., Li B., Yao F., Zheng X., Lin Q., Fang G. (2018). High-Performance Photodetectors
Based on Single All-Inorganic
CsPbBr3 Perovskite Microwire. ACS Photonics.

[53] Tong G., Jiang M., Son D.-Y., Ono L. K., Qi Y. (2020). 2D Derivative
Phase Induced Growth of 3D All Inorganic Perovskite Micro-Nanowire
Array Based Photodetectors. Adv. Funct. Mater..

[54] Zhang Y., Liu Y., Xu Z., Ye H., Li Q., Hu M., Yang Z., Liu S. F. (2019). Two-dimensional (PEA)_2_PbBr_4_ perovskite single crystals for a high performance
UV-detector. J. Mater. Chem. C.

[55] Gao T., Jiang Y., Yang S., Hu J., Zhang Z., Tang P., Cui Y., Sulaman M., Tang L., Zou B. (2023). Template-free synthesis of perovskite (PEA)_2_PbI_4_ nanowires by ion-intercalation processing for single-nanowire photodetectors. J. Alloys Compd..

[56] Zhou Y., Luo J., Zhao Y., Ge C., Wang C., Gao L., Zhang C., Hu M., Niu G., Tang J. (2018). Flexible Linearly
Polarized Photodetectors Based on All-Inorganic Perovskite CsPbI_3_ Nanowires. Adv. Opt. Mater..

[57] Tang X., Zhou H., Pan X., Liu R., Wu D., Wang H. (2020). All-Inorganic Halide Perovskite Alloy Nanowire Network Photodetectors
with High Performance. ACS Appl. Mater. Interfaces.

[58] Li J., Wang J., Ma J., Shen H., Li L., Duan X., Li D. (2019). Self-Trapped State Enabled Filterless
Narrowband Photodetections in 2D Layered Perovskite Single Crystals. Nat. Commun..

[59] Wang J., Li J., Lan S., Fang C., Shen H., Xiong Q., Li D. (2019). Controllable
Growth of Centimeter-Sized 2D Perovskite Heterostructures
for Highly Narrow Dual-Band Photodetectors. ACS Nano.

