# Supplementary material for: Two-Dimensional Perovskite Single-Nanowire Photodetectors
Source: ACS Photonics. 2026 Apr 20;13(9):2704–11. doi: 10.1021/acsphotonics.6c00352 (PMC13154357; doi:10.1021/acsphotonics.6c00352)
Supplement: Supplementary file 1 [file ph6c00352_si_001.pdf]

Supporting Information

Two-Dimensional Perovskite Single-Nanowire Photodetectors

Avija Ajayakumar,[a,b,c] *Jeong Hui Kim,* [a] *Nemanja Ninkovic,*[d] *Prashant Kumar,*[a] *Chakkooth Vijayakumar*[b,c] *Leonid Rokhinson,*[d] *Libai Huang,*[e] *and Letian Dou* [*a,e,f]

[a] Davidson School of Chemical Engineering, Purdue University, West Lafayette, IN 47907, United States
E-mail: letian.dou@emory.edu

[b] Chemical Sciences and Technology Division, CSIR-National Institute for Interdisciplinary Science and Technology (NIIST), Thiruvananthapuram 695 019, India

[c] Academy of Scientific and Innovative Research (AcSIR), Ghaziabad 201 002, India

[d] Department of Physics and Astronomy, Purdue University, West Lafayette, Indiana 47907, United States

[e] Department of Chemistry, Purdue University, West Lafayette, Indiana 47907, United States

[f] Department of Chemistry, Emory University, 1515 Dickey Drive, Atlanta, Georgia, 30322, United States

Number of pages: 5
Number of figures: 4 (Figures S1–S4)

Experimental Section:

Chemicals: Lead (II) bromide (Sigma–Aldrich, 99.99 %) and hydrobromic acid (Sigma–Aldrich, 48 wt% in H_2O), were used without further purification. TPA3-bromide was synthesized in the laboratory and used.

Perovskite nanowire growth: $(TPA3)_2PbBr_4$ single crystals were synthesized following a reported procedure, using either a *floating growth* or *slow cooling* approach. TPA3-bromide (0.4 mg) and $PbBr_2$ (20.3 mg) were dissolved in 300 µL aqueous HBr in a tightly sealed vial and heated (>100 °C with a heat gun or 160–180 °C on a hot plate) until a clear solution was obtained. All steps were performed in a fume hood.

For *floating growth*, the hot precursor solution was cooled to 50 °C and left undisturbed until precipitates appeared. A 1–3 µL aliquot of the supernatant was deposited onto a glass substrate, where nanocrystal nucleation occurred at the air–droplet interface. Crystals were collected on TEM grids, Si, Si/SiO_2, or PDMS stamps (GelPak), and excess solvent was removed with filter paper.

For *slow cooling*, the hot solution was placed in a Dewar containing boiling water and allowed to cool to room temperature over 72–96 h. Crystals were stored in the mother liquor until structural characterization.

Photodetector device fabrication: Titanium (Ti, 5 nm) was first deposited as an adhesion layer, followed by a 45 nm gold (Au) layer to form the electrodes, using electron-beam evaporation in a custom AJA deposition system. Subsequently, synthesized nanowires, were transferred onto these electrodes through a deterministic dry transfer method employing a polydimethylsiloxane (PDMS) stamp. In this process, the PDMS stamp, affixed to a glass slide, carries the nanomaterials and is positioned upside-down above the static device substrate. Precise alignment between the nanomaterials on the PDMS and the target electrodes on the substrate is achieved using a micromanipulator system under an optical microscope equipped with a digital camera. Once aligned, the PDMS stamp is gradually lowered until it makes gentle contact with the substrate, facilitating the transfer of nanomaterials onto the electrodes. If necessary, mild heating can be applied to the substrate to enhance adhesion and facilitate the transfer process. After maintaining contact for a few minutes, the PDMS stamp is slowly retracted, ensuring that the nanomaterials remain on the substrate.

Characterization:

All bright-field images were collected with a custom Olympus BX53 microscope equipped with a linear polarizer (U-AN360-3). All PL images were taken using an X-Cite Series 120 Q lamp as the excitation source. The filter cube in the Olympus BX53 microscope contains a 330–385 nm bandpass filter for

excitation. PL spectra were collected with a SpectraPro HRS-300 spectrometer. Single crystal data was collected on an instrument with kappa geometry, a Cu Kα wavelength (λ = 1.54178 Å) I-μ-S microsource X-ray tube, a laterally graded multilayer (Goebel) mirror for monochromatization, and a Photon III C14 area detector. Both instruments were equipped with an Oxford Cryosystems low temperature device and examination and data collection were performed at 150 KThe scanning electron microscopy (SEM) images were taken using a Hitachi S-4800 SEM at 5.0 kV with a secondary electron detector. Nanowire thickness was evaluated at three different positions across each sample using a surface profilometer (DekTak XT, Bruker, USA). The I-V measurements were performed using a Keithley 4200 source meter.

Calculation of device parameters:

Responsivity (R): R is the ratio of the photocurrent to P_{in} and can be calculated as

$$R = I_{ph}/(P_{in}*A)$$

and $I_{ph} = I_{light} - I_{dark}$

where I_{ph}, I_{light}, I_{dark}, P_{in}, and A are the photocurrent, current under light illumination, dark current, incident light intensity, and the effective device area, respectively.

Detectivity (D^*) reflects the ability of PDs to detect weak light signals. D^* is directly proportional to R, and inversely proportional to I_{dark}. D^* can be estimated using the following equation:

$$D^* = A^{1/2}*R/(2e*I_{dark})^{1/2}$$

where e is the elementary charge. This simplified approach is valid under typical operating conditions where shot noise from dark current serves as the dominant noise source, while other contributions such as Johnson noise and flicker noise remain relatively minor. These assumptions are particularly applicable for devices operating at room temperature under standard conditions.

EQE can be defined as the efficiency of PDs to convert incident photons to current, or simply the ratio between the output carriers and the number of incident photons. EQE can be given as:

$$\text{EQE}= Rhc/e\lambda$$

where h, c, e, and λ are the Planck constant, speed of light, electron charge, and wavelength of incident light, respectively.

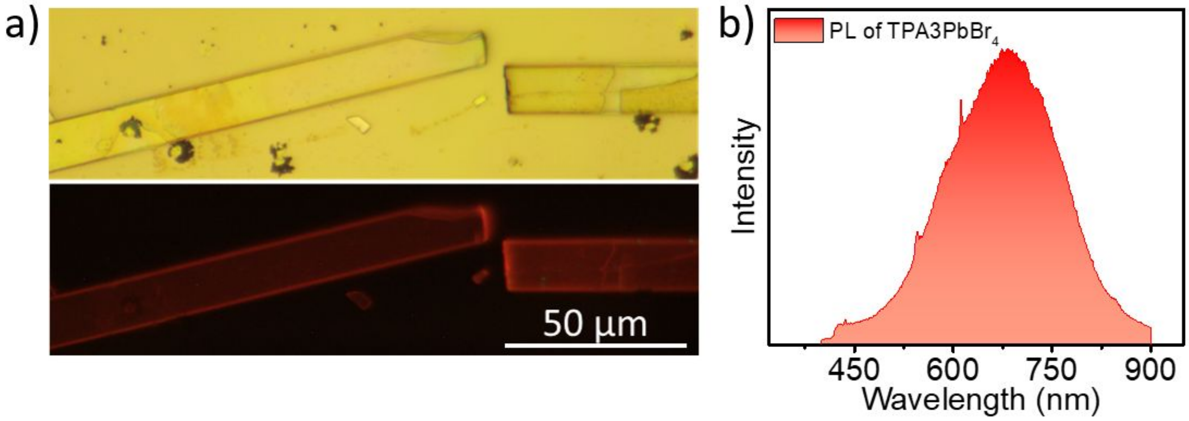

Figure S1: a) Microscopic image of the $(TPA3)_2PbBr_4$ nanowire under normal condition and under emission; b) steady-state PL spectra showing a broad excitonic peak.

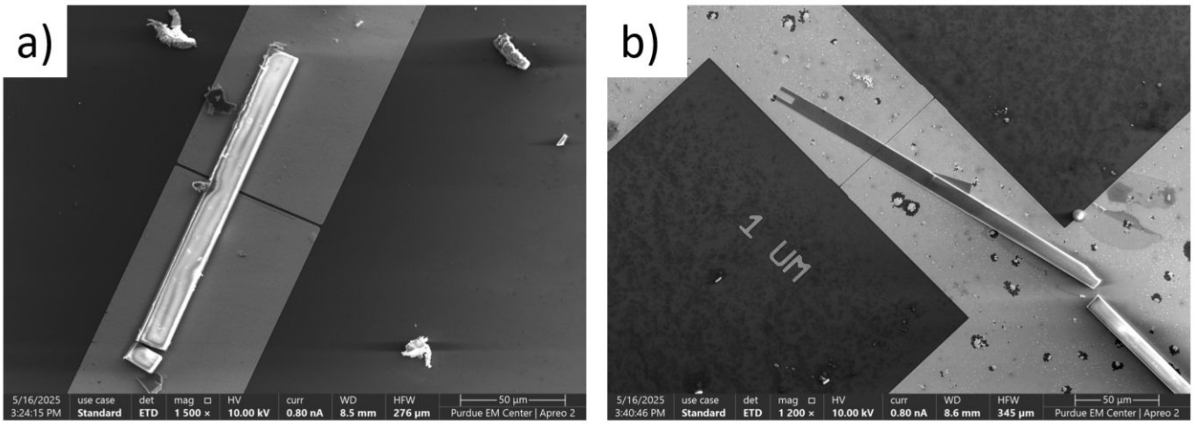

Figure S2: SEM images of devices with nanowire thicknesses of (a) 10 µm and (b) 4 µm.

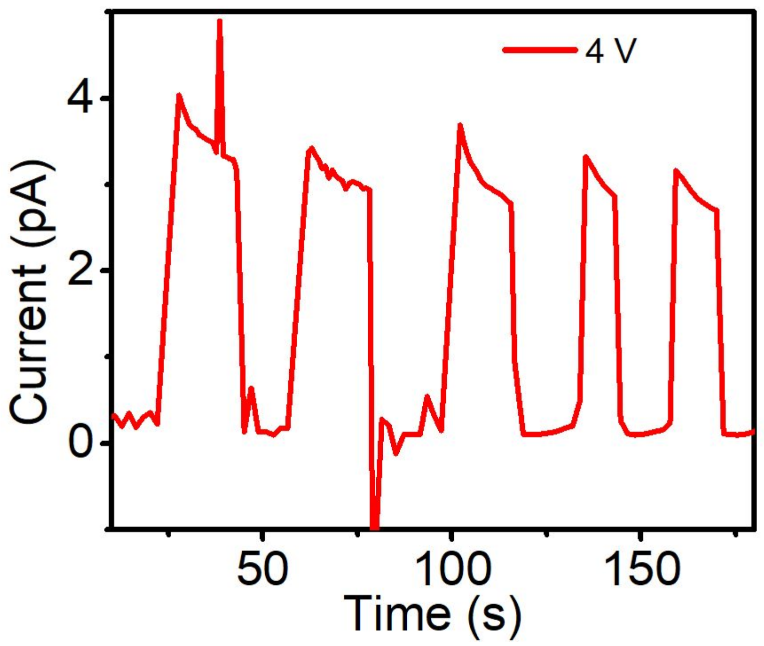

Figure S3: Photoresponse of $(TPA3)_2PbBr_4$ at a bias voltage of 4 V.

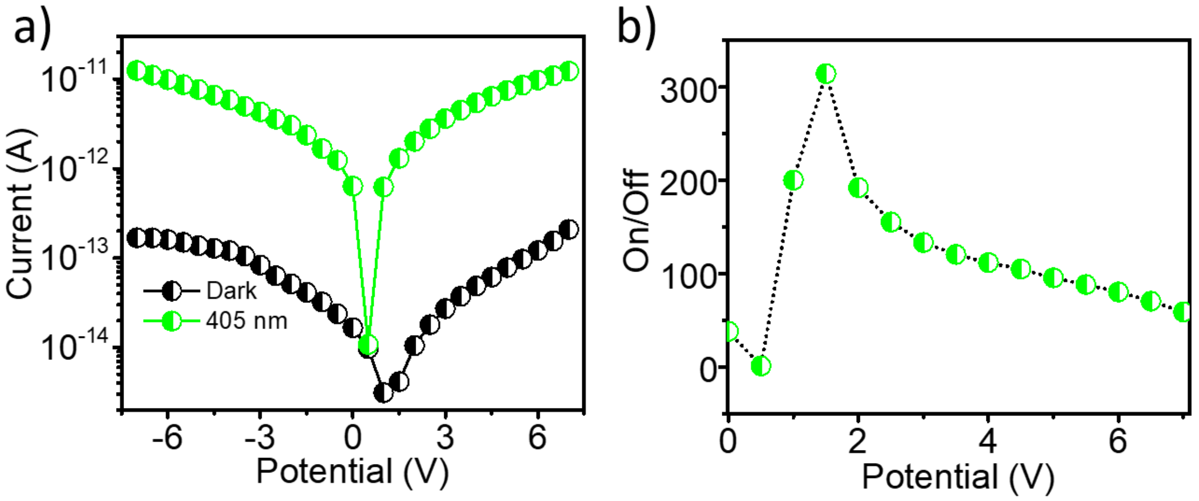

Figure S4: (a) I–V characteristics of the photodetector measured 40 days after fabrication; (b) corresponding bias-voltage-dependent on/off ratio.
